# Management of postoperative hypotony with fibrin glue following scleral tunnel-based explantation of pIOL and simultaneous ICL implantation: A case report

**DOI:** 10.1097/MD.0000000000047081

**Published:** 2026-01-09

**Authors:** Soo Han Kim, Si Hyung Lee, Eung Suk Lee

**Affiliations:** a Department of Ophthalmology, Ain Hospital, Ain Medical Foundation, Incheon, Republic of Korea; b Department of Ophthalmology, Soonchunhyang University Bucheon Hospital, College of Medicine, Soonchunhyang University, Bucheon, Republic of Korea.

**Keywords:** case report, fibrin glue, pIOL explantation, postoperative hypotony, scleral tunnel

## Abstract

**Rationale:**

Long-term implantation of anterior phakic intraocular lenses (pIOLs) can lead to progressive corneal endothelial cell loss, ultimately necessitating explantation. Although scleral tunnel approaches reduce corneal trauma during lens exchange, postoperative hypotony due to wound microleakage is an uncommon but vision-threatening complication. This report aims to present the 1st documented clinical case in which fibrin glue was successfully used to seal microleakage-induced hypotony following pIOL explantation and simultaneous toric implantable collamer lens (ICL) implantation.

**Patient concerns:**

A 36-year-old woman presented 12 years after bilateral Artiflex pIOL implantation with progressive corneal endothelial cell loss in the right eye (1886 cells/mm² vs 2612 cells/mm² in the left eye).

**Diagnoses:**

Corneal endothelial decompensation secondary to long-term anterior pIOL implantation, and early postoperative hypotony (intraocular pressure, 7 mm Hg) caused by aqueous leakage from a scleral tunnel incision.

**Interventions:**

The anterior pIOL was explanted via a 12 o’clock scleral tunnel (approximately 3.0 mm wide and 5–6 mm long) and replaced with a toric ICL using the soft-shell technique. Persistent postoperative hypotony was initially managed with resuturing but required fibrin glue application to definitively seal a microleak.

**Outcomes:**

After fibrin glue application, the intraocular pressure increased to 14 mm Hg the following day and remained stable throughout follow-up. At 1 month, the uncorrected visual acuity was 20/20, and corneal endothelial cell density stabilized at 1751 cells/mm² without further complications.

**Lessons:**

In cases where repeat suturing fails to control hypotony after scleral tunnel-based lens exchange, fibrin glue offers a minimally invasive, rapid, and effective alternative to restore wound integrity. Early detection of endothelial damage and the use of a low-trauma scleral tunnel approach can help preserve corneal health during pIOL-to-ICL transitions. However, as this is a single case, further evidence is needed to confirm the durability and safety of this approach in broader clinical settings.

## 1. Introduction

Refractive errors can be corrected by excimer laser procedures or phakic intraocular lens (pIOL) implantation. Among these, anterior pIOLs have been widely utilized as an effective option for myopia correction, as supported by previous clinical studies.^[1]^ However, long-term follow-up studies have shown that anterior pIOLs may cause a progressive reduction in corneal endothelial cell density as early as 3 years postoperatively,^[2]^ and severe endothelial loss has been reported in some cases, even after uneventful surgeries.^[3]^ Consequently, posterior chamber implantable contact lenses (ICLs) have largely replaced anterior pIOLs in the current practice. In this report, we present a case of significant corneal endothelial cell loss in the right eye 12 years after implantation of an Artiflex anterior pIOL (Ophtec), which led to explantation of the pIOL and implantation of a toric ICL (Aqua, STAAR Surgical). We further describe the development of postoperative hypotony and its successful management using fibrin glue.

## 2. Case presentation

A 36-year-old Korean woman presented to our clinic for the evaluation of corneal endothelial cell density and had undergone bilateral anterior pIOL implantation at a local clinic 12 years prior. She had no other notable ophthalmic or surgical history aside from lens implantation. At initial examination, her best-corrected visual acuity was 20/20 in both eyes (right eye: −1.25 Dsph, −1.25 Dcyl × 90°, left eye: −0.50 Dsph, −0.50 Dcyl × 120°). Slit-lamp biomicroscopy and fundus examination revealed no specific abnormalities, and intraocular pressure (IOP) was 18 mm Hg in both eyes. Specular microscopy revealed a significantly reduced corneal endothelial cell density of 1886 cells/mm² in the right eye compared with 2612 cells/mm² in the left eye. The central corneal thickness was 545 µm in the right eye and 546 µm in the left eye. After discussing the treatment options with the patient, we elected to explant the Artiflex pIOL from the right eye and replace it with a toric ICL.

The procedure was performed under topical anesthesia. Owing to preexisting endothelial cell loss in the right eye, a scleral tunnel, approximately 3.0 mm in width and 5 to 6 mm in length, was created at the 12 o’clock position instead of a corneal incision to minimize corneal trauma during the removal of the pIOL and implantation of the toric ICL. The tunnel was fashioned as a partial-thickness, self-sealing tract approximately 2 mm posterior to the limbus and directed into clear cornea, and there was no intraoperative evidence of wound gaping or obvious leakage at the end of surgery. To further protect the corneal endothelium, the soft-shell technique was employed using both cohesive and dispersive viscoelastic substances. Following lens implantation, the scleral incision was closed with 2 interrupted 10-0 nylon sutures and the conjunctival incision was closed with 3 interrupted 8-0 vicryl sutures. The patient was prescribed 0.5% moxifloxacin (Moxista; Sam Chun Dang Pharm, Seoul, Republic of Korea) and 1.0% prednisolone (Pred F; Lite PharmTech, Seoul, Republic of Korea) eye drops 4 times daily for postoperative care.

**Figure 1.**
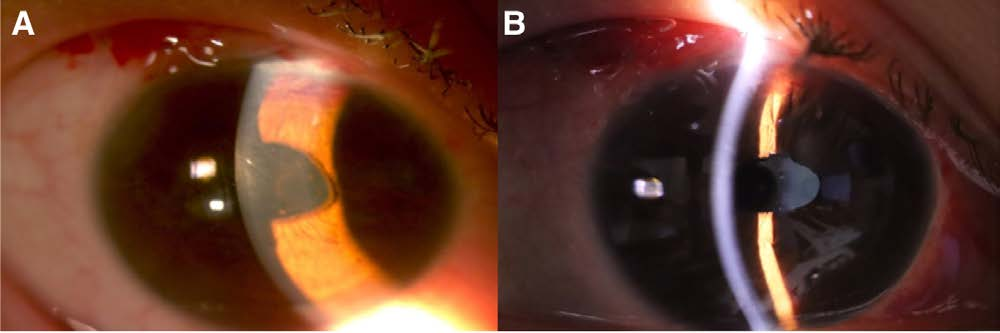
(A) Postoperative day 1 showing shallow anterior chamber and hypotony (intraocular pressure, 7 mm Hg) caused by aqueous leakage from the scleral tunnel. (B) Postoperative day 2 after fibrin glue application, showing restoration of anterior chamber depth and normalization of intraocular pressure (14 mm Hg).

**Figure 2.**
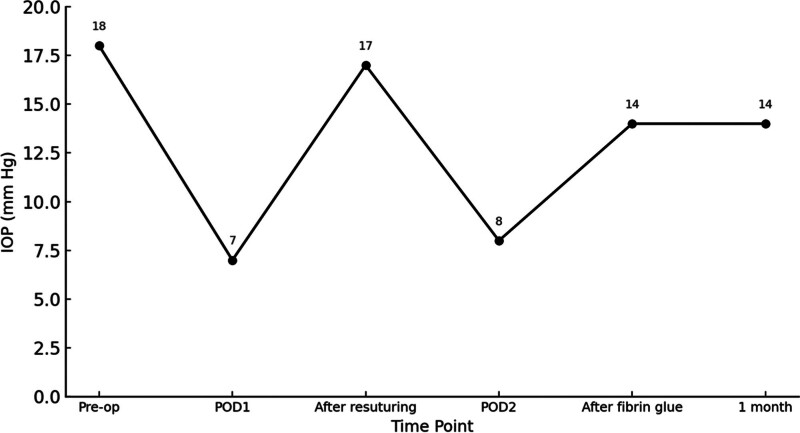
Course of intraocular pressure changes following scleral tunnel-based explantation of the anterior phakic intraocular lens and simultaneous toric ICL implantation, with stabilization after fibrin glue application. ICL = implantable collamer lens.

On the 1st postoperative day (POD), IOP in the right eye dropped to 7 mm Hg, suggesting aqueous leakage from the surgical site (Fig. 1A). Under local anesthesia, 2 additional interrupted 10-0 nylon scleral sutures were placed, bringing the total to 4 sutures, which temporarily increased the IOP to 17 mm Hg; the patient was then discharged. However, on POD 2, the IOP again decreased to 8 mm Hg. Persistent microleakage was suspected, despite additional suturing. Thus, it was determined that tissue adhesive would be more effective than repeated sutures in preventing further aqueous outflow. In the operating room, fibrin glue (Green Plast Q; Green Cross Corp., Gyeonggi-do, Republic of Korea) was applied along the edges of the conjunctival and scleral tunnels using a 25-gauge needle and maintained for 1 minute until solidification. The following morning, the IOP in the right eye was 14 mm Hg and remained stable thereafter without further hypotony (Fig. 1B). The overall course of IOP changes is also shown in Figure 2.

One month postoperatively, the uncorrected visual acuity in the right eye was 20/20, and the corneal endothelial cell density stabilized at 1751 cells/mm². The patient continued to undergo regular follow-up for endothelial cell count.

## 3. Discussion

This case illustrates the successful management of postoperative hypotony following intraocular lens exchange via a scleral tunnel approach using fibrin glue to effectively seal aqueous leakage and restore IOP. To our knowledge, no previous clinical report has documented the use of fibrin glue to treat hypotony caused by microleakage after scleral tunnel procedures. By promptly sealing microleaks that sutures may miss, this technique provides a novel therapeutic option with important clinical implications.

Postoperative hypotony may cause immediate complications, such as corneal edema and anterior chamber shallowing, and if persistent, may lead to severe long-term sequelae, including phthisis bulbi, choroidal detachment, optic disc swelling, and permanent vision loss.^[4]^ Because these complications can profoundly affect the visual prognosis, prompt and aggressive intervention is essential for managing hypotony.

The common causes of hypotony following scleral tunnel surgery include microleaks in the scleral tunnel, fistula formation at the conjunctival incision, ciliary body trauma or inflammation, and choroidal effusion. Subtle structural defects or microleaks in the scleral tunnel can lead to persistent aqueous leakage and require close attention. In this case, no ciliary body trauma was observed pre- or postoperatively; however, on POD 1, the anterior chamber had become shallow and the IOP had fallen to 7 mm Hg, strongly implying aqueous leakage through a micro-fistula and prompting immediate intervention.

In cases of mild hypotony, conservative treatments such as pressure patching or suppression of aqueous humor production may be considered. However, in this patient, the IOP dropped to 7 mm Hg and the anterior chamber depth was significantly reduced, prompting immediate resuturing. Although 2 additional interrupted 10-0 nylon sutures were placed, they did not completely prevent leakage. Repeated suturing may increase surgical site exposure and contact with instruments, thereby increasing the risk of infection and potential tissue damage. Moreover, because the patient was an active woman in her thirties, we anticipated a greater likelihood of fistula re-opening during daily activities; thus, a single definitive seal was preferred. To avoid these complications, fibrin glue is used as an alternative sealing method.

Fibrin glue has been employed in a variety of ophthalmic procedures to seal microleaks and provide additional fixation strength. It has been employed to stabilize enlarged scleral flaps during cataract surgery,^[5]^ secure intraocular lenses without sutures during repositioning procedures,^[6]^ and seal postoperative leaks after glaucoma surgery, thereby resolving hypotony.^[7]^

We performed a comprehensive literature search of PubMed/PMC, Google Scholar, and KoreaMed through September 14, 2025, using combinations of the terms “fibrin glue,” “fibrin sealant,” and “fibrin adhesive” with “scleral tunnel,” “scleral flap,” “hypotony,” “microleak,” “pIOL,” and “ICL implantation.” To our knowledge, no prior clinical reports specifically describing the use of fibrin glue to manage microleakage-induced hypotony after scleral tunnel-based pIOL explantation with simultaneous ICL implantation were identified. Representative studies have described fibrin glue use in related ophthalmic procedures, including sutureless posterior chamber IOL implantation,^[8]^ prevention of postoperative hypotony after glaucoma drainage surgery,^[9]^ sealing of filtering bleb leakage,^[10]^ and broader reviews summarizing its applications in ophthalmology.^[11]^ Although fibrin glue incurs a higher cost than repeat suturing, it offers clear advantages when leakage persists after resuturing or when surgery involves young, highly active patients. This reduces the need for repeated manipulation, lowers the risk of infection or additional tissue damage, and expedites recovery by providing a single definitive seal. Nevertheless, fibrin glue is known to be gradually resorbed within days to weeks, and rare adverse events such as local inflammatory or allergic reactions, as well as a theoretical risk of viral transmission from donor-derived products, have been documented in previous studies.^[11]^ In our case, the seal remained durable throughout follow-up without recurrence or complications, suggesting that fibrin glue may be a safe and effective adjunct in carefully selected patients.

Another important aspect of this case was the early detection of endothelial cell loss following anterior pIOL implantation and timely surgical response. The patient presented with significant endothelial cell reduction despite having no prior ocular trauma or inflammation and the postoperative stabilization of cell density supported anterior pIOL as the primary cause. Previous studies have identified mechanisms such as intraoperative mechanical trauma,^[12]^ elevated IOP,^[13]^ inflammatory reactions,^[14]^ and haptic–corneal contact^[15]^ as contributors to endothelial damage. In this case, the absence of trauma, inflammation, or haptic misalignment suggests intermittent contact between the pIOL and the corneal endothelium. Corneal endothelial cells lack regenerative capacity; thus, injury can lead to reduced cell density, polymegathism, and pleomorphism.^[16]^

It is generally recommended to consider lens explantation when the density falls below 2000 cells/mm², with immediate removal advised if the count is <1500 cells/mm².^[17]^ In this case, the endothelial cell count declined to 1886 cells/mm², prompting our decision to remove and replace the lens.

The use of a small-incision scleral tunnel and soft-shell technique effectively minimized corneal trauma, and the postoperative endothelial cell count (1751 cells/mm²) remained relatively stable. This surgical strategy may offer a safe and effective approach for transitioning from anterior to posterior chamber lenses in patients with endothelial compromise.

This case report has several limitations. First, it describes a single clinical case with short- to mid-term follow-up, which limits the ability to determine long-term durability of fibrin glue sealing in this setting. Second, assessment of the scleral tunnel structure and wound integrity relied on slit-lamp examination without adjunctive imaging such as anterior segment optical coherence tomography. Therefore, while the outcome was favorable in this case, the findings may not be fully generalizable to all patients undergoing scleral tunnel-based lens exchange.

## 4. Conclusion

This case report describes the successful management of postoperative hypotony following intraocular lens exchange via a scleral tunnel using fibrin glue to effectively seal aqueous leakage and stabilize IOP. Given that hypotony can cause rapid shallowing of the anterior chamber and corneal edema in the short term, leading to serious long-term complications, such as choroidal detachment and optic nerve damage, early intervention is critical. In this case, despite repeated suturing attempts, the leakage persisted and was controlled by the application of fibrin glue. This represents a novel therapeutic approach that has not been previously reported in the literature and may offer a valuable alternative for managing post-surgical hypotony.

Additionally, early detection of corneal endothelial cell loss after anterior pIOL implantation allows for timely surgical intervention. The use of a small-incision scleral tunnel and soft-shell technique enabled safe transition to a posterior chamber ICL while minimizing further endothelial damage. This method may serve as an effective surgical strategy for addressing the complications associated with long-standing anterior chamber phakic lenses.

## Author contributions

**Conceptualization:** Eung Suk Lee.

**Investigation:** Eung Suk Lee.

**Project administration:** Eung Suk Lee.

**Supervision:** Eung Suk Lee.

**Writing – original draft:** Soo Han Kim.

**Writing – review & editing:** Si Hyung Lee.
